# Neural Stem Cells in the Diabetic Brain

**DOI:** 10.1155/2012/820790

**Published:** 2012-11-14

**Authors:** Tomás P. Bachor, Angela M. Suburo

**Affiliations:** Medicina Celular y Molecular, Facultad de Ciencias Biomédicas, Universidad Austral, Buenos Aires, B1629AHJ Pilar, Argentina

## Abstract

Experimental diabetes in rodents rapidly affects the neurogenic niches of the adult brain. Moreover, behavioral disorders suggest that a similar dysfunction of the neurogenic niches most likely affects diabetic and prediabetic patients. Here, we review our present knowledge about adult neural stem cells, the methods used for their study in diabetic models, and the effects of experimental diabetes. Variations in diet and even a short hyperglycemia profoundly change the structure and the proliferative dynamics of the neurogenic niches. Moreover, alterations of diabetic neurogenic niches appear to be associated with diabetic cognitive disorders. Available evidence supports the hypothesis that, in the adult, early changes of the neurogenic niches might enhance development of the diabetic disease.

## 1. Old and New Neurogenic Niches in the Adult Brain

Adult neurogenesis has been known since the 1960s [1]. Although the two main neurogenic niches, the dentate gyrus (DG) of the hippocampus and the telencephalic subventricular zone (SVZ), were described in those years, intensive studies only began in the 1980s [2]. Research has been mainly done in rodents [3], but there is good evidence of neurogenesis throughout life in the human DG [4, 5] and SVZ [6–10]. A recent review exhaustively describes the properties of these niches in human beings [11]. 

### 1.1. Neurogenesis in the Dentate Gyrus

**Figure 1:**
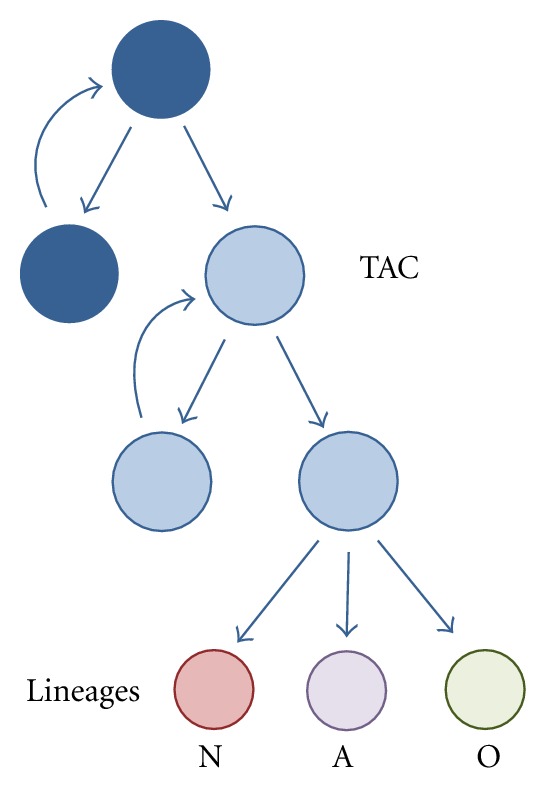
The dynamics of proliferation and differentiation in the adult neurogenic niches. Neural stem cells (NSCs) divide, both symmetrically and asymmetrically for self-renewal and production of transit amplifying cells (TACs). The term neuroprogenitors encompasses TACs and true NSCs. TACs rapidly divide symmetrically to amplify the availability of neural and glial precursors and finally differentiate into one of the three neural lineages: neuronal (N), astroglial (A), or oligodendroglial (O).

In all mammalian species, the DG and the SVZ share a lineage including neural stem cells (NSCs) (or primary stem cells), transit amplifying cells (TACs) (or secondary stem cells), and newborn cells of the three final phenotypes: neuronal, astroglial, and oligodendroglial (Figure 1). By contrast, each niche shows a specific topographical arrangement and characteristic dynamics for cell proliferation, differentiation, and migration. These features are reflected in the different terminology used for each niche. In the DG, NSCs are called type 1 cells whereas TACs are included in type 2. Postmitotic immature neurons are type 3 cells. Type 1 precursors express the glial markers glial fibrillary acidic protein (GFAP) and brain lipid binding protein (BLBP), together with Sox2 and Nestin but do not display the calcium-binding protein S-100*β*. The latter is expressed by postmitotic astrocytes [12, 13]. Type 1 cells are self-renewing and multipotent cells, but they seldom divide [14]. Type 2 cells are morphologically different from type 1 cells, since they have short horizontally oriented processes. The presence of GFAP in type 2 cells has been denied by early studies [12, 15], but supported by more sensitive tests [16]. Type 2 cells also express Sox2 and Nestin, and display the polysialylated form of neural cell adhesion molecules (PSA-NCAM) [10, 17–19]. Some authors distinguish two developmental stages, types 2a and 2b, depending on the appearance of the cytoskeletal protein doublecortin (DCX), an immature neuronal marker [12]. Type 3 cells are described as postmitotic immature neurons that express markers of the neuronal lineage (PSA-NCAM, DCX, NeuroD, Prox1) and lack glial markers [12]. Type 3 cells might undergo proliferation, as in the case of seizures [20]. About half of the generated cells survive and integrate into existing neuronal circuits [21]. Dietary modulation and physical exercise may regulate cell proliferation in the hippocampus [22]. Although many questions remain, it is generally accepted that hippocampal newborn neurons are involved in the establishment of spatiotemporal relationships among multiple environmental cues and would be required for allocentric (not egocentric) space representation, long-term memory retention, and flexible inferential memory expression [23]. Moreover, new DG neurons seem to be necessary for flexibility in some specific parameters of learning [24]. 

### 1.2. Neurogenesis in the Subventricular Zone

In the SVZ, NSCs also form a special subpopulation of GFAP-expressing cells (B1 cells) that originates TACs and proliferating neuroblasts (C cells) [25–29]. Most B1 cells contact the ventricle through small, specialized apical processes that contain a single primary cilium [30, 31]. The identity of true NSCs in the SVZ is still controversial [32, 33], since activated CD133+ ependymal cells might also serve as adult NSCs [34, 35]. NSCs can be identified *in vitro* by their capacity to form free-floating aggregates (neurospheres) and the clonal differentiation of the three lineages [36]. *In vivo*, they can be distinguished from TACs by their slow cell cycle [25, 37, 38]. TACs are also described as intermediate progenitor cells [28]. Together with differentiating cells they migrate through the rostral migratory stream (RMS) to the olfactory bulb, where they generate at least seven types of inhibitory interneurons. Most NSCs descendants follow the neuronal lineage [39], probably because bone morphogenetic protein (BMP) signaling within the neurogenic niches determines a neurogenic fate [40]. Proliferating cells can be labeled by the minichromosome maintenance protein 2 (mcm2) [37], which accumulates during the G1 phase of cycling cells, and the antigen Ki-67, a nuclear protein present in every cell except those in the resting phase [17]. Nestin is an intermediate filament expressed by neurogenic precursors at different stages of differentiation [39]. Niche cells also express endothelins, particularly after brain injury [35, 41].

In the adult, myelinating oligodendrocytes are continuously produced from local oligodendrocyte precursor cells (OPCs) residing in the brain parenchyma [42]. Nevertheless, subventricular type B cells can generate a small number of OPCs and mature oligodendrocytes that migrate laterally and dorsally into the corpus callosum, the fornix, and the fiber tracks of the striatum. Subventricular oligodendrogenesis increases after demyelination of the corpus callosum [43–45]. The perinatal SVZ is a secondary source of brain astroglia [46], but only a small proportion of adult-born cells differentiate into mature astrocytes (GFAP/S100*β*) [47]. Astroglial cells follow a particular migration pathway in the coronal plane, migrating out of the SVZ into the dorsal white matter and cortex, the striatum, and, through the lateral migratory stream (LMS), into the lateral white matter and cortex [46]. Migrating astrocytes give rise to a reservoir of cortical multipotent cells [48]. In addition, mature astrocytes proliferate locally generating large numbers of postnatal astrocytes [49]. 

Proliferation in the SVZ can be regulated by ablation of the olfactory epithelium or exposure to an enriched olfactory environment [27]. Dietary restriction, of great benefit in diabetic patients [50, 51], increases resistance of the SVZ and the DG to excitotoxic injury [52]. Endocannabinoids would be involved in these dietary effects [53]. Large modifications of the SVZ occur after brain injury, when cells derived from this niche migrate to the striatum or the telencephalic cortex. There, they mainly form cells expressing oligo- and astroglial markers [35, 41, 54–56].

### 1.3. Neurogenesis in the Hypothalamus

Cell proliferation and neuron differentiation in the hypothalamus have been reported in rodents and other mammals [57–59]. Neural stem cells have been identified in the wall of the third ventricle [59], the median eminence [60], and the arcuate nucleus [61]. 

The rat hypothalamus shows low levels of proliferation, but administration of IGF-1 significantly increases the number of proliferating cells [59]. Hypothalamic neurogenesis can also be modulated by external temperature [58]. In the murine arcuate nucleus, neurons involved in energy-balance regulation exhibit substantial turnover, with more than half of them being replaced between 4 and 12 weeks of age [62]. Genetically obese mice with leptin deficiency (*ob*/*ob* mice) lack hypothalamic NSCs [63]. Moreover, proliferation is suppressed in high-fat-diet-induced obesity, in part due to increased apoptosis of newly divided cells [63]. In the median eminence, tanycytes fulfill the role of NSCs. Tanycytes are hypothalamic radial glia-like ependymal cells that generate newborn neurons associated with weight and metabolic regulation. After feeding rats with a high-fat diet, neurogenesis in the median eminence quadruples that of control animals [60]. Available evidence suggests that hypothalamic neurogenesis contributes to the control of energy balance in response to environmental changes [61]. Nutritional status might control energy balance circuits by selective generation of specific neuronal subtypes that promote or inhibit feeding, combined with the elimination of neurons with opposing functions by selective apoptosis [60].

## 2. Diabetes and the Brain: The Problem and the Models

### 2.1. The Problem

Diabetes mellitus refers to a group of chronic metabolic conditions associated with abnormally high levels of blood glucose. The disease follows two main courses: type 1 (T1DM), characterized by loss of insulin producing cells, and type 2 (T2DM), reflecting peripheral resistance to insulin, sometimes with reduced insulin secretion. The latter is the most frequent clinical presentation and afflicts about 24 million people in the United States and about 285 million people worldwide [64]. It is estimated that 13% of the adult US population suffers from this condition, whereas another 30% has prediabetes (defined as impaired glucose tolerance, impaired fasting glucose, or both) [65]. The increased frequency of diabetes is correlated with a parallel increase in obesity [66]. 

From a clinical perspective, diabetes appears as a cardiovascular disease [67]. Typical end-organ disease affects kidneys, eyes, and the peripheral nervous system. T2DM is associated with a 1.5–2.5-fold increased risk of dementia including Alzheimer's disease [68, 69]. Diabetic patients also show a characteristic cognitive dysfunction that can be explained by the combination of vascular lesions with neuroinflammatory brain disease [70]. However, recent evidence shows that neuronal dysfunction appears in the retina before major vascular alterations [71]. 

Both diabetic cognitive impairment and Alzheimer's disease are correlated with alterations of the hippocampus [72, 73], one of the two neurogenic niches of the adult brain. Altered hippocampal neurogenesis is also present in mood disorders [72], which are frequently found in diabetic and prediabetic patients [74]. There is no direct evidence of the role of human neurogenic niches in the diabetic cognitive deficit. However, the effects of cognitive stimulation, meditation, exercise, and antidepressant treatment in prevention of the hippocampal atrophy associated with age and diabetes have been associated to activation of neurogenesis [75]. 

### 2.2. Neurogenesis in Diabetic Rodents

Damage to the insulin-producing cells of the pancreas using streptozotocin (STZ) [76] remains one of the most used models for induction of diabetes. A high-fat diet is another frequently used approach to induce obesity and insulin resistance in C57Bl6 mice [77]. In addition, diabetes appears in a chronic fashion in several murine and rat strains [78]. Some of these models show the biochemical blood profile and pathogenesis of T2DM [79, 80]. The Animal Models Section of the NIDDK-sponsored Diabetic Complications Consortium currently displays an update of frequently used rodent models [81].

Most experimental studies of the diabetic neurogenic niches attempt to identify changes in the proliferation, differentiation, and survival of neuroprogenitors. The standard procedure is labeling of DNA-synthesizing cells with the thymidine analog 5-bromodeoxyuridine (5-BrdU) [82]. Immediate detection of labeled cells provides an estimation of proliferation rate, whereas label retention after one week or more reflects differentiation and survival. Ki-67 is also a widely used marker for proliferating cells [17]. Differentiation can be evaluated by the appearance of the neuroblast markers DCX and PSA-NCAM or neuronal markers such as *β*-III-tubulin and Neu-N [82]. Double labeling is useful to measure the amount of 5-BrdU-incorporating cells that survive to differentiate into neurons or glia [17].

Early studies done in the rat DG, 2 days after STZ, revealed a fourfold decrease in the number of proliferating (5-BrdU-labeled) cells [83]. Neurogenesis impairment in the STZ-diabetic mice can be reverted by oestradiol [82] or the antidepressant fluoxetine [84], both in the hippocampus and the SVZ. Oestradiol and fluoxetine were given 10 days after diabetes induction and had no effect on blood glucose levels, showing that progenitors are still present and can proliferate even in the presence of significant hyperglycemia [82, 84]. Experimental diabetes also reduces the number of surviving neurons. It has been estimated that neuronal production at 7 weeks after diabetes induction is less than 20% of control levels [17]. In the non-obese diabetic (NOD) mice, a model for T1DM, glial activation appears in the hippocampus during the prediabetic stage [85], together with a decrease in the survival of 5-BrdU-labeled nuclei [86]. 

Cell proliferation in the DG is also decreased in the Zucker diabetic fatty rat. In this T2DM model, the DG contains a smaller number of newly differentiated neurons, with shorter dendrites, than controls [87]. Neurogenesis is always higher in nondiabetic rats, even after blood glucose levels have been normalized by exercise [18, 87]. In another T2DM model, the Goto-Kakizaki rat, the DG niche shows an increase of proliferation together with a decrease in survival of newly born neurons [88, 89]. 

Information about hypothalamic neurogenesis in diabetic animals is not available. However, it has been reported that astrocytes in the hypothalamus and other brain and spinal regions of diabetic rodents are reduced in number, both due to increased apoptosis and reduced proliferation [90–93]. 

### 2.3. Physiopathology in the Diabetic Neurogenic Niche

The precise mechanisms that mediate dysfunction of the neurogenic niches are presently unknown. In addition, diabetic consequences on neurogenesis possibly change according to the magnitude of the metabolic derangement. Thus, decrease of neurogenesis correlates with the increase in blood glucose levels (Bachor, unpublished). In addition, diabetes courses with chronic inflammation and oxidative stress that may independently promote neuronal death and inhibit neurogenesis [94, 95]. Hyperglycemia could be a direct causal factor, since alterations of neurogenesis are detected just 2 days after streptozotocin injection [83]. Moreover, neurogenesis deficits occur not only after insulin depletion but also after increased insulin resistance, as in the *db*/*db* mice [96] and the Goto-Kakizaki rats [89]. 

After they become hyperglycemic, Goto-Kakizaki rats show a higher progenitor proliferation rate than age-matched WKY rats. By contrast, the number of neural progenitors surviving for 3 weeks is significantly lower in the diabetic than in the control rats [89]. Neurosphere cultures obtained from Goto-Kakizaki SVZ stop growing much earlier than those from control SVZ. Those derived from the diabetic DG survive as many passages as those from control DG. However, they do not respond to FGF2- and IGF1, two neurotrophic factors that stimulate growth of control neurospheres [89]. 

Diabetic lipotoxicity is another factor potentially associated to neurogenesis impairment. Culture of SVZ-derived NSCs in the presence of 0.2–0.3 mM palmitate, which mimics the hyperlipidemic milieu, triggers proapoptotic mechanisms. Under these conditions, activation of the pituitary adenylate cyclase-activating polypeptide (PACAP) receptors promotes survival of NSCs. These receptors are expressed in the adult SVZ, and their levels are higher in *ob*/*ob* mice than in control mice [97]. 

## 3. Diabetes and the Hypothalamic-Pituitary-Adrenal Axis (HPA)

The HPA axis is activated in people with type 2 diabetes, and increased cortisol concentrations have been linked with cognitive impairment [98]. Increased corticosterone is also found in insulin-deficient STZ rats and insulin-resistant *db*/*db* mice, two models that also show impairment of hippocampus-dependent memory and neurogenesis. In these mice, adrenalectomy plus low corticosterone replacement reverses the changes of hippocampal function and cell proliferation [96]. 

In addition, corticosterone-dependent inhibition of neurogenesis appears in several stress models [99]. Stress generally decreases cell proliferation, an effect that can be reproduced by glucocorticoid administration [100]. Paradoxically, physical exercise and housing in an enriched-environment stimulate neurogenesis and neuron survival both in control and in diabetic rats [87, 101]. However, both experimental paradigms also activate the HPA axis and increase circulating glucocorticoid levels. It has been suggested that these contradictory effects might be explained by the rewarding nature of exercise and the enriched environment. Rewarding social experiences release factors such as endogenous opioids, oxytocin, dopamine, brain-derived neurotrophic factor (BDNF), and insulin-like growth factor, which would protect against elevated glucocorticoids [99]. 

On the other hand, the hypothalamus is one of the primary brain sites that senses information on the body's nutritional status and responds with suitable behavioral and metabolic responses that maintain energy homeostasis [102]. Rats and mice consuming a high-fat diet have elevated corticosterone levels and impaired hippocampal neurogenesis [103]. After seven weeks of high fat diet, mouse hippocampi show a decrease of 5-BrdU-labeled cells, without changes in GFAP o DCX labeling, and reduced BDNF levels [104]. By contrast, dietary restriction in rats and mice promotes neurogenesis by inducing the survival and differentiation of newly generated neurons together with an increase of BDNF [105]. 

Of note, other mechanisms are probably involved. Thus, ingestion of sucrose and fructose solutions reduces neurogenesis through a corticosterone-independent pathway, possibly related to apoptosis mediated by elevated TNF-*α* levels in the circulation [106].

## 4. Antidiabetic Drugs Enhance Neurogenesis

The incretins Glucagon Peptide-1 (GLP-1) and Gastric Inhibitory Polypeptide (GIP) are gut-derived hormones released in response to an ingested meal. Incretins regulate postprandial phenomena: they enhance insulin release from pancreatic beta cells, inhibit postprandial glucagon secretion, and block gastric emptying, thus reducing blood glucose. Released GLP-1 is short-lived, since it is rapidly hydrolyzed by the enzyme dipeptidyl-peptidase-IV. Exendin-4 (exenatide) and liraglutide are GLP-1 ligands used in the treatment of T2DM. The GLP-1 receptor is expressed in the SVZ of adult rodents, and its activation by exendin-4 stimulates cell proliferation and differentiation *in vivo* and *in vitro* [107]. In nondiabetic mice, exendin-4 administration during 21 days increases the number of Ki67-, DCX-, and 5-BrdU+DCX-immunoreactive cells in the DG, compared to the control group [108]. Exendin-4 also enhances progenitor cell division in different diabetic mouse models, whereas a GLP-1 receptor antagonist reduces progenitor cell proliferation [109]. Similarly, liraglutide increases expression of the proneural gene Mash1 in *ob/ob* mice. Mice deficient in the GIP receptor have a lower amount of neuroprogenitor cells [110], whereas a GIP receptor agonist increases neuroprogenitor proliferation [111].

Metformin, a widely used antidiabetic drug, promotes neurogenesis and enhances spatial memory formation through the activation of a signaling pathway essential for neurogenesis [112]. Interestingly, metformin affects some metabolic pathways also affected by dietary restriction, such as AMP-activated protein kinase (AMPK). This kinase is a metformin main target that regulates glucose homeostasis, lipid metabolism, and gluconeogenesis [113]. Metformin also activates the atypical protein kinase C (aPKC), which is downstream of AMPK. This enzyme is required to bypass the block imposed by insulin resistance through phosphorylation of the transcriptional coactivator CREB-binding protein (CBP) [114]. The aPKC/CPB pathway is not only crucial to reduce the expression of gluconeogenic enzymes, but is also essential to generate neurons from neuron precursors. Metformin can activate the neurogenic pathway in the adult mouse *in vivo*, enhancing spatial memory formation [112].

## 5. Neurogenesis, Blood Vessels, and Stroke

Adult NSCs have a close relationship with the vascular system [115]. In the adult SVZ, stem cells make direct contact with the endothelium at sites lacking astrocyte end-feet [29]. Thus, NSCs would be directly exposed to blood-borne molecules, including glucose or lipids. Endothelial cells recruit activated NSCs and TACs by chemotaxis, which is regulated by stromal-derived factor 1 (SDF1) and CXC chemokine receptor 4 (CXCR4) [116]. Endothelial cells also produce betacellulin (BTC), an EGF-like growth factor. The latter, however, acts mainly on TACs, whereas BTC receptors are present on neuroblasts and progenitor cells [117]. Thus, diabetic disturbance of endothelia [118] could have a direct effect on adult NSCs.

Experimental stroke is accompanied by increased activity of neurogenic niches [35, 55, 56, 119]. A similar activity has been detected in poststroke human cortex [120], and it has been postulated that SVZ neuroprogenitors have a role in brain repair during disease [11]. Therefore, in a diabetic patient, the increased risk for premature and severe stroke [121] would be aggravated by dysfunction of adult neurogenic niches, predicting weakened repair mechanisms. 

Interestingly, in middle-aged diabetic Goto-Kakizaki rats, exendin-4 treatment during 4 weeks before and 2 weeks after experimental stroke significantly increases neuron survival, as shown by the count of Neu-N-positive cells [122]. In addition, exendin-4-treated animals show a 2-fold greater number of proliferating cells plus a 1.5-fold increase of neuroblasts in the SVZ as compared with the PBS group [122]. These effects were observed with low exendin-4 doses that did not reduce glycemia. 

## 6. Diabetes and Neurodegenerative Diseases

Adult neurogenesis is deteriorated in Alzheimer's disease (AD) models and precedes neuronal loss. Moreover, molecules involved in AD pathogenesis, such as presenilins and amyloid precursor protein, also play important roles in neurogenesis [123]. Epidemiological studies link T2DM to (AD) and vascular dementia [68, 124], suggesting a common pathogenic factor. Neurogenesis could be involved, since T2DM and these neurological diseases share a relationship with insulin receptor and GLP-1 neuroprotective effects [125]. Study of autopsy material has shown that increasing AD severity associates with progressively reduced levels of mRNAs for insulin, IGF-I, and IGF-II polypeptides and their receptors [126]. Available evidence supports the notion that brain glucose uptake and utilization are impaired in AD [127]. Failing insulin/IGF1 signaling contributes, through diverse mechanisms, to neurodegeneration [128]. Regions most affected by AD (hippocampus, temporal lobe, and diencephalon) seem to be those with the largest expression of insulin and IGF receptors [127]. Findings about the effects of metformin on adult neurogenesis suggest that a similar therapeutic approach could be beneficial both for diabetes and Alzheimer's disease [112]. However, a recent epidemiological study did not provide evidence to link long-term metformin treatment with a reduction in AD risk. Rather, it suggested a greater risk [129].

Diabetes may also be considered a risk factor for future Parkinson's disease (PD) [130]. The risk elevation seems largely limited to individuals who had had diabetes for more than 10 years at the time of the survey [131], indicating that vascular disease and hypoglycemia events may contribute to this association. Damage to neurogenic niches might occur at early stage, previous to the appearance of typical motor symptoms [72]. Such damage could perhaps be associated with diabetes or insulin resistance.

## 7. Conclusions

The hypothesis that impaired neurogenesis is one of the main factors involved in the diabetic cognitive deficit is strongly supported by experimental evidence obtained in rodent models, together with strong anatomopathological and epidemiological findings in human beings. Both T1DM and T2DM impair cell proliferation and neuron differentiation in the main neurogenic niches of the adult brain, the DG, and the SVZ. This association is explained by abundant physiopathologic evidence. However, the proportion of cognitive deficit and dementia that can be ascribed to neurogenesis impairment has still to be determined. Human diabetic cognitive deficit is not yet an early, diagnosis and by the time it is usually recognized that brains have most likely accumulated a large load of oxidative and vascular damage. 

On the other hand, impairment of adult neurogenic niches might explain the progressive course of T2DM [132]. Early alteration of neurogenic niches might mislead brain sensing of their nutritional status and facilitate the appearance of metabolic changes. Most likely, people that will later show T2DM have suffered previous changes of their neurogenic niches. Techniques are becoming available for the study of human neurogenic niches [133], allowing noninvasive testing [134] of this hypothesis.
